# Supplementary material for: Selective ensembles in supported palladium sulfide nanoparticles for alkyne semi-hydrogenation
Source: Nat Commun. 2018 Jul 6;9:2634. doi: 10.1038/s41467-018-05052-4 (PMC6035185; doi:10.1038/s41467-018-05052-4)
Supplement: Supplementary file 1 — Supplementary information [file 41467_2018_5052_MOESM1_ESM.pdf]

Supplementary Information

Selective ensembles in supported palladium sulfide nanoparticles for alkyne semi-hydrogenation

Albani et al.

Supplementary Note 1 Additional characterization of the catalysts. Comprises **Supplementary Table 1** and **Supplementary Figures 1-5**.

Supplementary Table 1 Synthesis conditions and characterization data of the catalysts.

Catalysts	$T_{sulfidation}$ / K	Pd[a] / wt.%	S/Pd[b] / mol mol^{-1}	S_{BET}[c] / m^2 g^{-1}	V_{pore}[d] / cm^3 g^{-1}	d_{Pd}[e] / nm	D_{CO}[f] / %
Pd_3S/C_3N_4-373	373	0.44	0.41	267	0.76	1.8	41
Pd_3S/C_3N_4-398	398	0.42	0.91	260	0.62	2.0	35

[a]ICP-OES; [b]S content by elemental analysis; [c]BET method; [d]Volume of N_2 adsorbed at $p/p_0 = 0.95$; [e]TEM image analysis of 100 particles; [f]CO chemisorption.

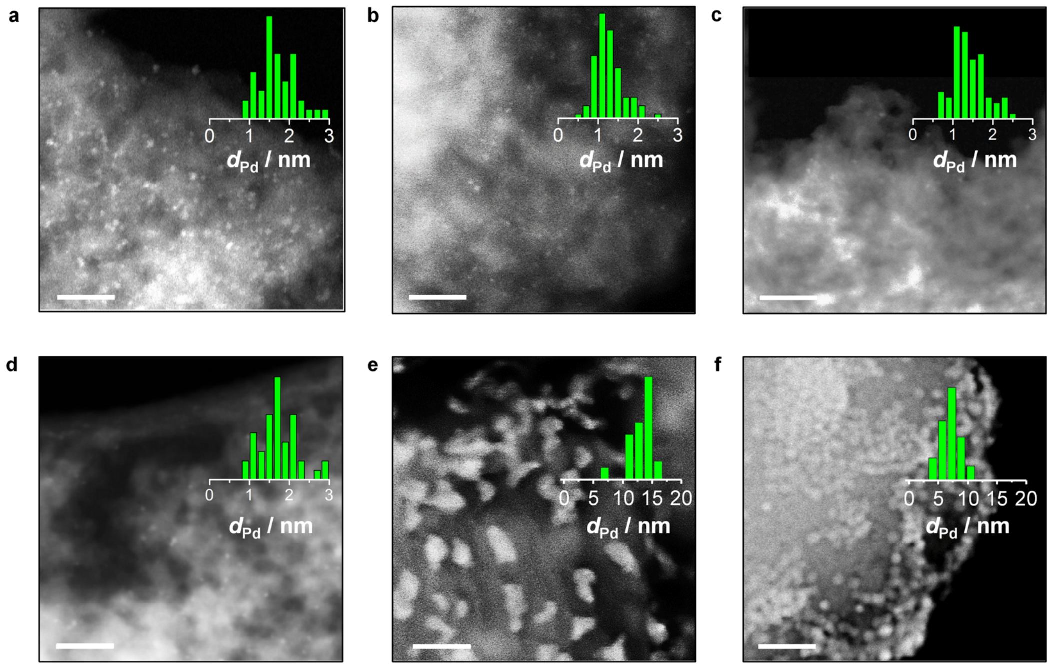

Supplementary Figure 1 High-angle annular dark field scanning transmission electron microscopy images of **a** $Pd_xS@C_3N_4$-423, **b** Pd/C_3N_4, **c** Pd_3S/C_3N_4-373, **d** Pd_3S/C_3N_4-398, **e** $PdPb/CaCO_3$, and **f** Pd-HHDMA and the derived particle size distributions of the Pd-containing nanoparticles (inset). Scale bars: 30 nm.

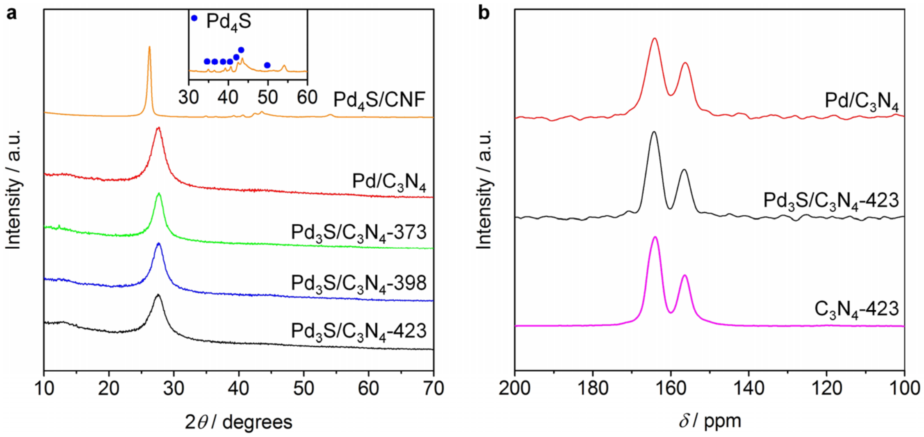

Supplementary Figure 2 a X-ray diffraction patterns confirm the preserved crystalline structure of the carbon nitride host, the prominent broad reflection at around 27.3° 2θ corresponds to the graphite-like interlayer stacking. The absence of additional reflections associated with the palladium nanoparticles or their sulfided analogues over C_3N_4 is consistent with the relatively low metal content and high dispersion observed for these phases. The Pd_4S/CNF sample shows a characteristic diffraction line at 25.2° 2θ corresponding to the (002) graphitic plane, and reflections due to the Pd_4S phase as highlighted in the inset. **b** ^{13}C magic-angle spinning nuclear magnetic resonance spectra of the catalysts and the host upon sulfidation (C_3N_4-423) confirm the stability of the host to the respective post-synthetic modifications. The two main signals at 164 and 157 ppm are attributed to the $CH_2(NH_x)$ and CN_3 moieties, respectively.

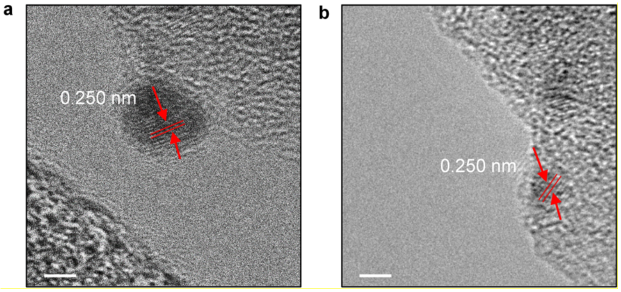

Supplementary Figure 3 High-resolution transmission electron micrographs of **a** Pd_3S/C_3N_4-373, and **b** Pd_3S/C_3N_4-398. Regularly-spaced lattice fringes were observed over all of the palladium-containing nanoparticles with interplanar distances: close to 0.250 nm in all cases. Scale bars: 2 nm.

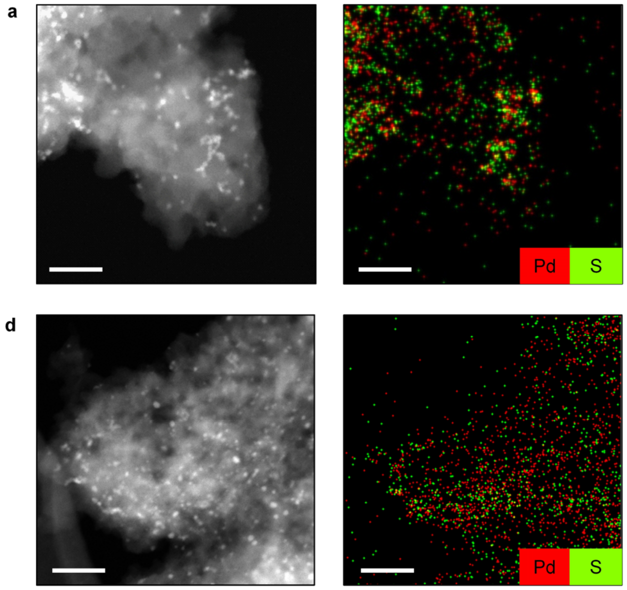

Supplementary Figure 4 High-angle annular dark field scanning transmission electron microscopy images and corresponding energy-dispersive X-ray spectroscopy maps of Pd_3S/C_3N_4-423 **a** fresh and **b** after 50 h on stream. Scale bars: 30 nm.

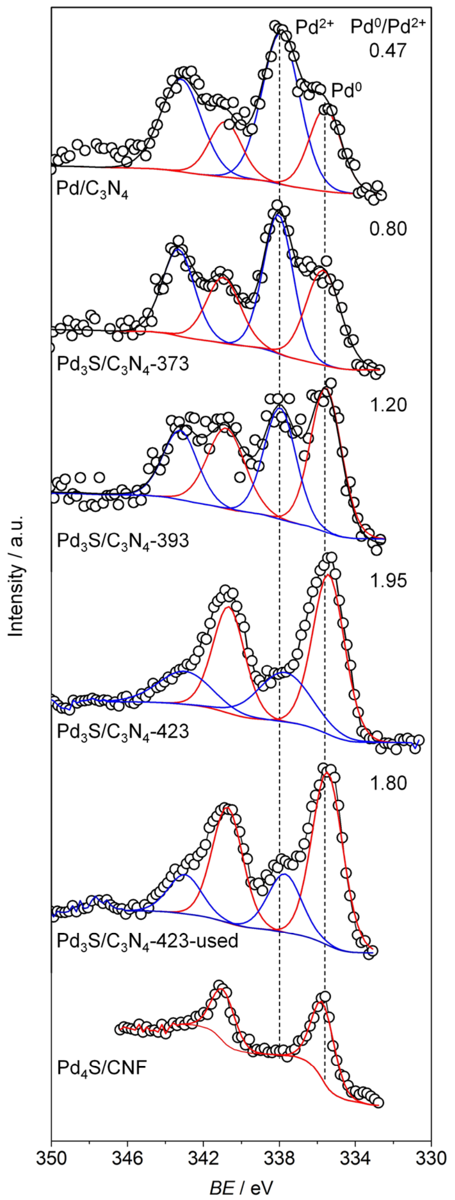

Supplementary Figure 5 Pd 3*d* core level X-ray photoelectron spectra of Pd/C_3N_4, Pd_3S/C_3N_4-373, Pd_3S/C_3N_4-398, Pd_3S/C_3N_4-423 fresh, Pd_3S/C_3N_4-423 after 50 h on stream, and Pd_4S/CNF. Solid black lines show the results of fitting the raw data (circles), the red and blue lines correspond to the individual peaks after deconvolution. The dashed lines indicate the positions of the identified metal species (Pd^0 and Pd^{2+}), the ratio of which is shown.

Supplementary Note 2 Catalytic testing including kinetic analyses. Comprises **Supplementary Figures 6-10**, **Supplementary Tables 2-3**, and related discussion.

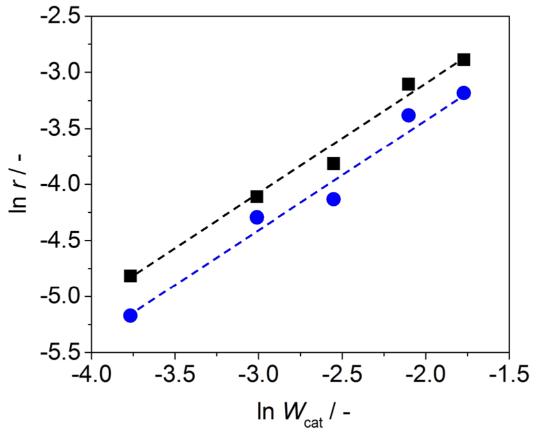

Supplementary Figure 6 Reaction rate as a function of the amount of catalyst at different temperature $T = 303$ and 323 K (blue and black, respectively). Conditions: $P = 1$ bar, F_L(2-methyl-3-butyn-2-ol+toluene) = 1.0 cm^3 min^{-1}, and $F_G(H_2)$ = 36 cm^3 min^{-1}. The fitted line features a slope of *ca.* 1, which confirms that the reaction proceeds under kinetic control. The similar dependence at both temperatures excludes the presence of heat-transfer limitations.

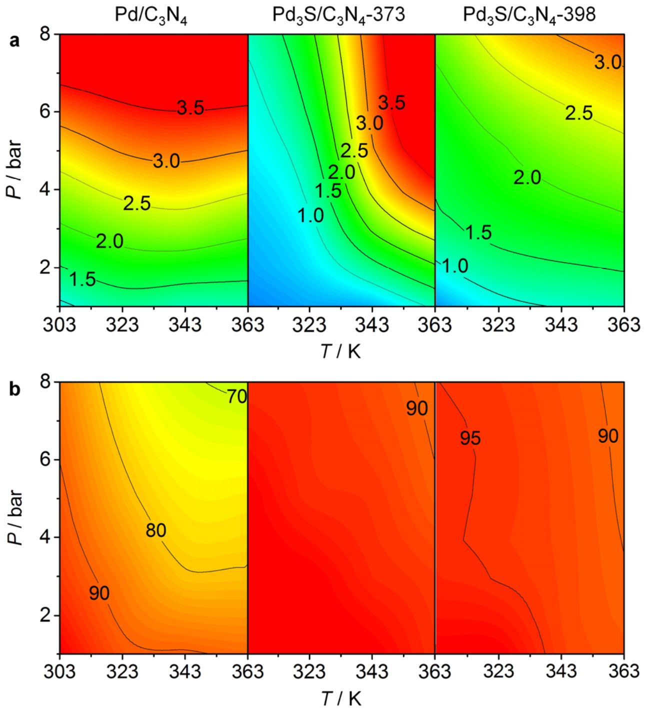

Supplementary Figure 7 a Reaction rate (in 10^3 h^{-1}) and **b** selectivity to 2-methyl-3-buten-2-ol (in %) as a function of temperature and pressure in the hydrogenation of 2-methyl-3-butyn-2-ol over selected catalysts. Conditions: $W_{cat} = 0.1$ g, F_L(2-methyl-3-butyn-2-ol+toluene) = 1.0 cm^3 min^{-1}, and $F_G(H_2) = 36$ cm^3 min^{-1}. The contour maps were obtained through spline interpolation of 14 experimental points.

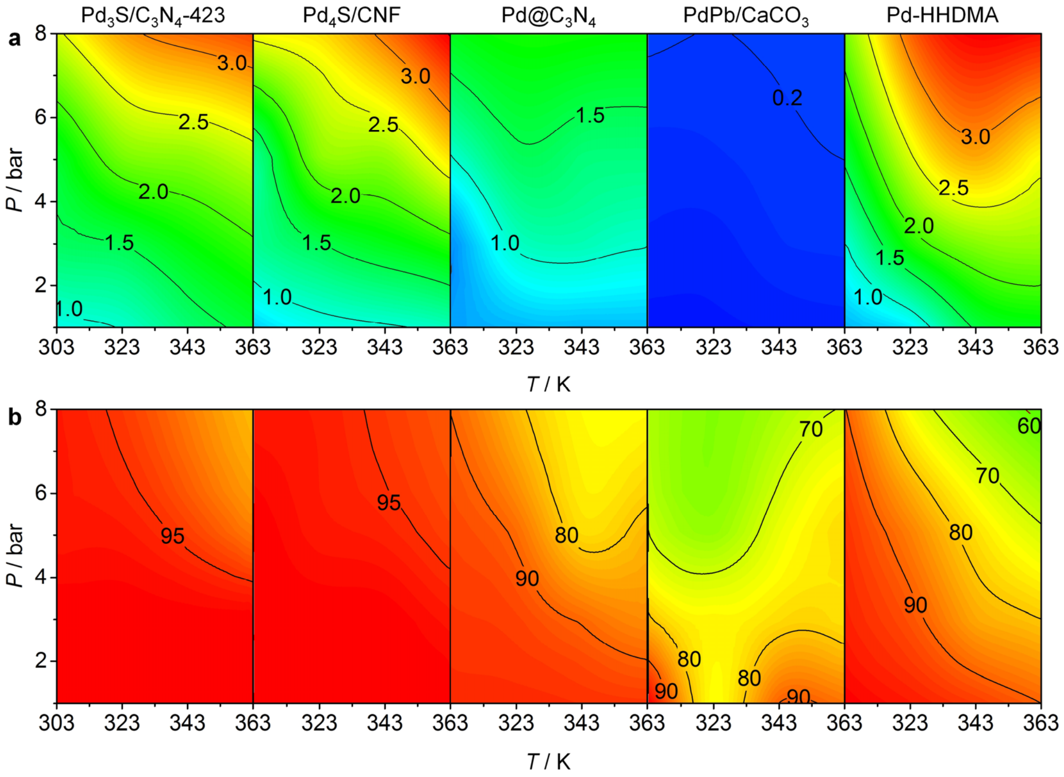

Supplementary Figure 8 a Reaction rate (in 10^3 h^{-1}) and **b** selectivity to 1-hexene (in %) as a function of temperature and pressure in the hydrogenation of 1-hexyne over selected catalysts. Conditions: $W_{cat} = 0.1$ g, F_L(1-hexyne+toluene) = 1.0 cm^3 min^{-1}, and $F_G(H_2) = 36$ cm^3 min^{-1}. The contour maps were obtained through spline interpolation of 14 experimental points.

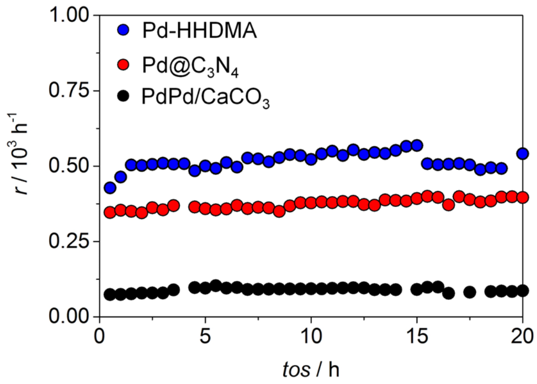

Supplementary Figure 9 Stability of the state-of-the-art catalysts in the hydrogenation of 2-methyl-3-butyn-2-ol to 2-methyl-3-buten-2-ol. Conditions: $W_{cat} = 0.1$ g, $T = 303$ K, $P = 1$ bar, F_L(2-methyl-3-butyn-2-ol+toluene) = 1.0 cm^3 min^{-1}, and $F_G(H_2) = 36$ cm^3 min^{-1}.

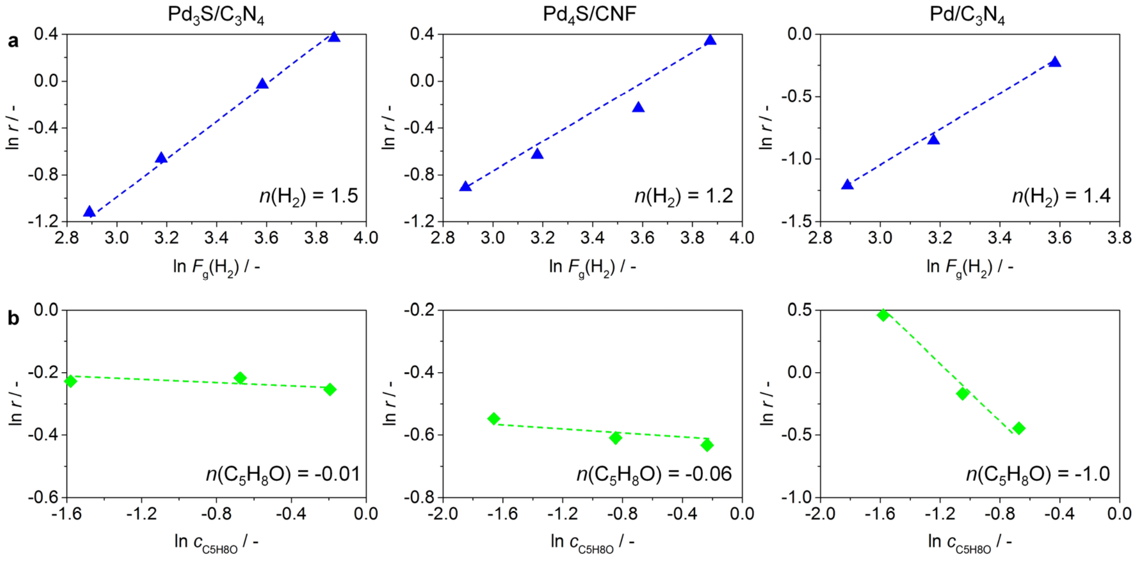

Supplementary Figure 10 Reaction rate as a function of the inlet partial pressure of **a** H_2, and the concentration of **b** 2-methyl-3-butyn-2-ol (c_{C5H8O}) over Pd_3S/C_3N_4, Pd_4S/CNF, and Pd/C_3N_4. As expected, all of the catalysts display a similar dependence on p_{H_2}, but the Pd_xS catalysts show a distinct trend compared to the unmodified palladium with c_{C5H8O}. Conditions: W_{cat} = 0.1 g, T = 303 K, P = 1 bar.

Considering the DFT-calculated reaction profiles, the competing/non-competing (single- or dual-site mechanism) character of the reactants on the surface, and the presence of the solvent, two different reaction pathways can be derived and are summarized in **Supplementary Table 2** (where * and □ are two different sites on the surface of the catalyst).

As the most energy demanding process, step 4 is considered as rate determining, and consequently the rate can be written as:

$$r = k_2 \theta_{C_5H_8O} \theta_H \quad \textbf{Eq. 1}$$

Assuming that steps 2 and 3 are in *quasi*-equilibrium and that step 1 is bound by saturation:

$$c_{H_2} = \frac{sKp_{H_2}}{1 + Kp_{H_2}} \quad \textbf{Eq. 2}$$

where c_{H_2} is the concentration of hydrogen in the solvent (toluene), p_{H_2} is the hydrogen gas pressure, s is the solubility of hydrogen in the solvent, and K is the corresponding equilibrium constant.

Using the aforementioned assumptions, the obtained rates for the two different mechanisms, and the corresponding reaction orders (n) are reported in **Supplementary Table 3**. From the rate equation and corresponding reaction orders, we can observe that the bifunctional mechanism ensures that both

reactants are bound to the surface on different sites. Note that it also occurs on $Pd_4S(200)$ due to the facile migration of the activated H fragments to the S atoms forming thiol groups.

Supplementary Table 2 The elementary steps of the single- and dual-site mechanism over the Pd-based catalysts.

Step	Description	Single site	Dual site
1	H_2 solution	$H_2(g) \rightarrow H_2(sol)$	$H_2(g) \rightarrow H_2(sol)$
2	H_2 adsorption	$H_2(sol) + 2* \rightarrow 2H*$	$H_2(sol) + 2\square \rightarrow 2H\square$
3	Alkyne adsorption	$C_5H_8O(sol) + * \rightarrow C_5H_8O*$	$C_5H_8O(sol) + * \rightarrow C_5H_8O*$
4	First H transfer	$C_5H_8O* + H* \rightarrow C_5H_9O* + *$	$C_5H_8O* + H\square \rightarrow C_5H_9O*$
5	Second H transfer	$C_5H_9O* + H* \rightarrow C_5H_{10}O + 2*$	$C_5H_9O* + H\square \rightarrow C_5H_{10}O + * + \square$

Supplementary Table 3 Kinetic derivations for the single- and dual-site mechanisms.

	Mechanism	
Description	Single site	
Site balance	$1 = \theta * + \theta_{H} + \theta_{C_5H_8O}$	**Eq. 3**
Rate	$r = k_4 K_3 \frac{\sqrt{K_2\, c_{H_2}}}{\left[1 + K_3 c_{C_5H_8O} + \sqrt{K_2 c_{H_2}}\right]^2} c_{C_5H_8O}$	**Eq. 4**
$n(H_2)$	$n(H_2) = \frac{\partial \ln(r)}{\partial \ln(p_{H_2})}$	1.5
$n(C_5H_8O)$	$n(C_5H_8) = \frac{\partial \ln(r)}{\partial \ln(c_{C_5H_8O})}$	–1
Description	Dual site	
Site balance	$1 = \theta^{\square} + \theta_{H}$	**Eq. 5**
	$1 = \theta * + \theta_{C_5H_8O}$	**Eq. 6**
Rate	$r = k_4 K_3 \frac{\sqrt{K_2 c_{H_2}}}{\left[1 + K_3 c_{C_5H_8O}\right]\left[1 + \sqrt{K_2 c_{H_2}}\right]} c_{C_5H_8O}$	**Eq. 7**
$n(H_2)$	$n(H_2) = \frac{\partial \ln(r)}{\partial \ln(p_{H_2})}$	1.5
$n(C_5H_8O)$	$n(C_5H_8) = \frac{\partial \ln(r)}{\partial \ln(c_{C_5H_8O})}$	0

Supplementary Method 1 Full computational details.

Density Functional Theory (DFT) calculations with periodic boundary conditions[1] were performed using the Vienna *Ab initio* Simulation Package (VASP)[2,3] employing the revised Perdew-Burke-Ernzerhof (RPBE) functional[4]. The interaction between the valence and the core electrons was described with the projected augmented wave (PAW) method[5]. A plane-wave cutoff energy of 450 eV was used for the valence electrons. In all calculations, van der Waals contributions were expressed using the semi-empirical DFT-D2 approach[6,7] and the parameters were refined for palladium in our group[8]. The calculated lattice parameters for the primitive orthorhombic bulk unit cell of Pd_3S, optimized using a dense Monkhorst Pack of 6×6×6 *k*-point mesh (a_{calc} = 6.18, b_{calc} = 5.45, and c_{calc} = 7.55 Å), agree well with the experimental data (a_{exp} = 6.09, b_{exp} = 5.37, and c_{exp} = 7.45 Å)[9]. The simulated lattice parameters for the primitive bulk cell of Pd_4S using 8×8×8 *k*-point mesh are a_{calc} = 5.20, and c_{calc} = 5.70 Å, while the experimental values are a_{exp} = 5.12, and c_{exp} = 5.59 Å[10]. The calculations of surface energy for Pd_3S were carried out for various terminations of several low-index planes, namely, (001), (010), (100), (110), (202), (011), and (111), as well as (402) and (504) that are open planes, using periodic boundary conditions. The Pd_3S surfaces were constructed with six S-Pd-S trilayers, separated by a vacuum space of 14 Å, where the two topmost S-Pd-S trilayers were fully relaxed, whereas the four bottommost layers were fixed to their bulk positions. The results on Pd_3S surfaces calculations can be found in **Supplementary Note 3**. The low hydrogen and alkyne binding energies on the Pd_xS surfaces together with the operation in three-phase conditions limit the likelihood of surface modification in the presence of the reactants. Therefore, differently from simulations on the gas-phase alkyne semi-hydrogenation coverages are small and they do not perturb the materials to induce new phases (*i.e.*, hydrides)[21].

To compute the reaction mechanism and gain a molecular-level understanding, acetylene is taken as a representative molecule. To demonstrate the validity of the approach, key calculations were conducted for the adsorption of 2-methyl-3-butyn-2-ol and 2-methyl-3-buten-2-ol (*vide infra*). Multiple pathways occurring during acetylene hydrogenation, namely semi-hydrogenation, complete hydrogenation, and oligomerization, were investigated on Pd(111), and two of most stable configurations of Pd_3S surfaces: Pd_3S(001) and Pd_3S(202). For comparative purposes, the Pd_4S system was also investigated. In

particular, two different crystal terminations were considered, the (110) surface reported to be the most stable by Miller *et al.*,[11] and the (200) facet that our experimental and theoretical results indicate is the most relevant. The Pd(111) surface was modeled as a periodically repeating *p*(3×2) slab consisting of five atomic layers, where the three at the bottom were fixed and the two on the top were fully relaxed, and the lowest-energy structure was found using a (4×6×1) *k*-point mesh. On the other hand, the Pd_3S(001) and Pd_3S(202) surfaces were simulated as periodically repeated *p*(2×1) and *p*(1×1) slabs, respectively, consisting of six S-Pd-S trilayers, and refined using a Γ-centered (3×6×1) *k*-point mesh. The Pd_4S(110) and Pd_4S(200) surfaces were modeled using *p*(1×1) and *p*(2×1) slabs using (6×4×1) and (3×6×1) *k*-point mesh. The adsorption of atomic sulfur (S_s@Pd(111)) and its inclusion into subsurface sites (S_{ss}@Pd(111)) on the Pd(111) surface were also computed. For these calculations, the Pd(111) surface was built using a slab featuring a 3×3 surface unit cell with five atomic layers, and optimized using *k*-point 4×4×1 grids. To study C-C coupling reactions (oligomerization) and the adsorption of larger molecules (2-methyl-3-butyn-2-ol, and 2-methyl-3-buten-2-ol), bigger supercells *p*(3×4), *p*(2×2), *p*(1×2), *p*(2×2), and *p*(2×1) were used for the Pd(111), Pd_3S(001), Pd_3S(202), Pd_4S(200) and Pd_4S(110) surfaces, respectively, and optimized using a (4×3×1), (3×3×1), (3×3×1), (3×3×1), and (3×4×1) *k*-point meshes, respectively. The climbing image-modified nudged elastic band (CI-NEB) method was used to assess the activation energies of the different reaction paths[12,13]. The nature of all reaction minima and transition states was confirmed by means of numerical frequency analyses obtained from the Hessian calculated with displacements of 0.015 Å.

The use of the energy profiles instead of the Gibbs energies is deemed a good approximation since (i) the solvent contributions to entropy are still under discussion, (ii) solvent replacement energies would be more relevant, (iii) the zero-point vibrational energy (ZPVE) and vibrational contributions for competing steps are comparable for similar molecules, thus the effect on the energy barriers for their transformation cancels out (**Supplementary Table 6**). Also, although the computed adsorption energies are small, there is a significant difference in the entropic contributions between gas- and three-phase operation, and the values obtained for adsorption confirm that the solvent molecules can be replaced by the reactants in an efficient manner (**Supplementary Table 8**).

The adsorption energy of C_2H_2 and C_2H_4, the activation energy for H_2 dissociation, the ensemble area, and the segregation energy for different catalytic structures (CeO_2(111), PdPb(111), PdZn(111), Ag(211), Au_{19}, Pd(111)-HHDMA, and Pd@C_3N_4) have also been calculated to derive a *vis-à-vis* comparison with Pd_xS (**Fig. 7**). The PdPb(111), PdZn(111), Pd(111)-HHDMA, and Ag(211) surfaces were modeled with five atomic layers, where the three at the bottom were fixed and the two on the top were fully relaxed, consisting of a *p*(2×2), *p*(1×1), *p*(3×3), and *p*(2×1) surface unit cell using (6×8×1), (6×6×1), (3×3×1) and (6×6×1) *k*-point mesh, respectively. The CeO_2(111) surface was modeled with four O-Ce-O trilayers, and refined using a *Γ*-centered (5×5×1) *k*-point mesh. The Pd@C_3N_4 was built with four C_3N_4 layers consisting of *p*(2×2) supercells using (1×1×1) *k*-point mesh. The same calculations were performed on the Au_{19} cluster in a box of 17×15×12 Å with a single *k*-point.

Supplementary Note 3 Structure of palladium sulfide. Comprises **Supplementary Table 4**, **Supplementary Figures 11-16**, and related discussion.

According to the binary Pd-S phase diagram, five crystallographically stable structures have been identified of different stoichiometry including PdS (*P4₂/m*), PdS_2 (*Pbca*), Pd_4S (*P42₁c*), Pd_3S (*Ama2*), and $Pd_{16}S_7$ (*I43m*)[14]. Among these, Pd_3S has an orthorhombic crystal structure belonging to the space group *Ama2* (number 40), which contains 12 Pd and 4 S atoms in the primitive bulk cell. Using the SUBGROUPGRAPH computer program available on the Bilbao Crystallographic Server[15] the space group *Ama2* is found to be linked to the Pd space group *Fm3m via* different possible paths or chains. To analyze the degree of ionic or covalent character of the Pd_3S crystal, the electron localization function (ELF) for this system has been evaluated along the (100) and (001) planes and is plotted in **Supplementary Figure 11a**. The results show that the maximum ELF value between Pd and S atoms is greater than 0.8, consistent with covalent bonding. To further discern the bonding character between Pd and S atoms, the difference in charge density (*i.e.*, the crystal density minus the superposition of isolated atomic densities) of the Pd_3S crystal and the q_e (total charge) per atom transfer from Pd to S by the Bader charge analysis have been calculated[16]. The former shows charge accumulation mainly between Pd and S atoms (**Supplementary Figure 11b**), while the latter reveals an average electron transfer of 0.1 from each Pd to S, resulting in a net gain of 0.3 electrons on S due to the stoichiometry

of the crystal. The results from the ELF, difference charge density, and Bader analyses, point to classifying Pd_3S as a covalent crystal with a small charge transfer from Pd to S atom.

Surface terminations of Pd_3S crystal

For a given model slab with Miller indices (*hkl*), the surface energy, γ_s, is defined by the following expression:

$$\gamma_s = \frac{1}{2A}\left[E_{\text{slab}}^{\text{unrelaxed}} - NE_{\text{bulk}}\right] + \frac{1}{A}\left[E_{\text{slab}}^{\text{relaxed}} - E_{\text{slab}}^{\text{unrelaxed}}\right] \quad \textbf{Eq. 8}$$

where A is area of the surface considered, $E_{\text{slab}}^{\text{unrelaxed}}$ and $E_{\text{slab}}^{\text{relaxed}}$ are the respective total energies of the unrelaxed and relaxed slabs, N is the number of atoms in the slab, and E_{bulk} the total energy of the bulk per atom, and the factor 1/2 accounts for the two surfaces in the slab unit-cell. Notice that, due to the asymmetric configuration of the slab the relaxation contribution only affects one of the two surfaces. Surface energy calculations were carried out on 9 different possible surfaces of the Pd_3S crystal and their respective planes featuring different Miller indices (*hkl*) are illustrated in **Supplementary Figure 12**. The results, listed in **Supplementary Table 4**, indicate that S-terminated surfaces are more stable than their unmodified Pd-terminated counterparts, which is in line with the termination stability reported for Pd_4S surfaces[11]. It is clear that the (001) facet is the most stable with a surface energy of 0.65 J m^{-2}.

Supplementary Table 4 The lattice parameters, *k*-point sampled, and calculated energies, for different Pd_3S surface facets.

Facet (*hkl*)	Lattice parameter / Å *a*	*b*	*k*-point	γ_s / J m^{-2}
001	6.19	5.46	6×6×1	0.65
010	7.55	6.19	5×6×1	1.07
100	5.46	7.55	6×5×1	0.85
110	7.55	8.26	5×4×1	1.13
101*	9.76	5.46	3×6×1	0.80
011	6.19	9.32	6×3×1	1.06
111	8.26	9.94	4×3×1	1.38
402	16.32	5.46	2×6×1	0.91
504	45.15	5.46	1×6×1	0.99

*equivalent to 202

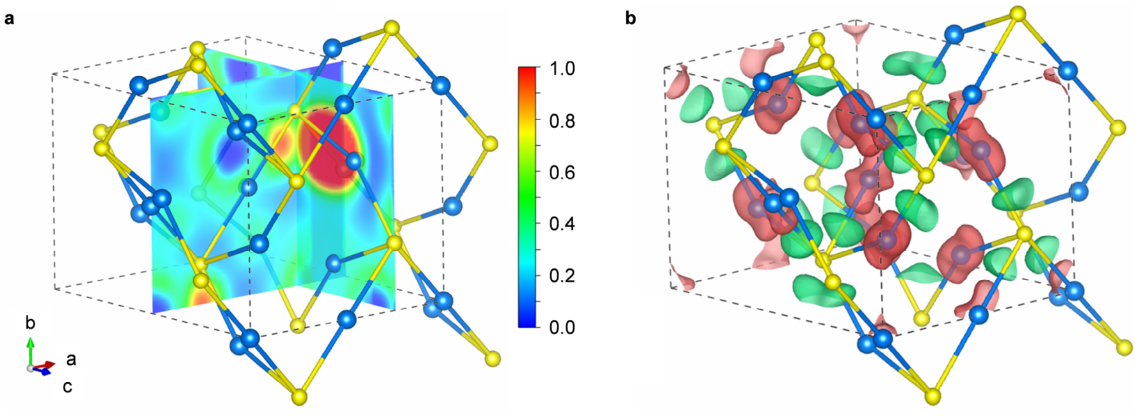

Supplementary Figure 11 **a** Electron localization function (ELF) maps and **b** the difference charge density (*i.e.*, the crystal density minus the superposition of isolated atomic densities) in a single unit cell of Pd_3S. The green and red isosurfaces in **b** represent the maximum and minimum values at 0.01, and −0.025 a_0^{-3}, respectively. Color code: palladium (blue), sulfur (yellow).

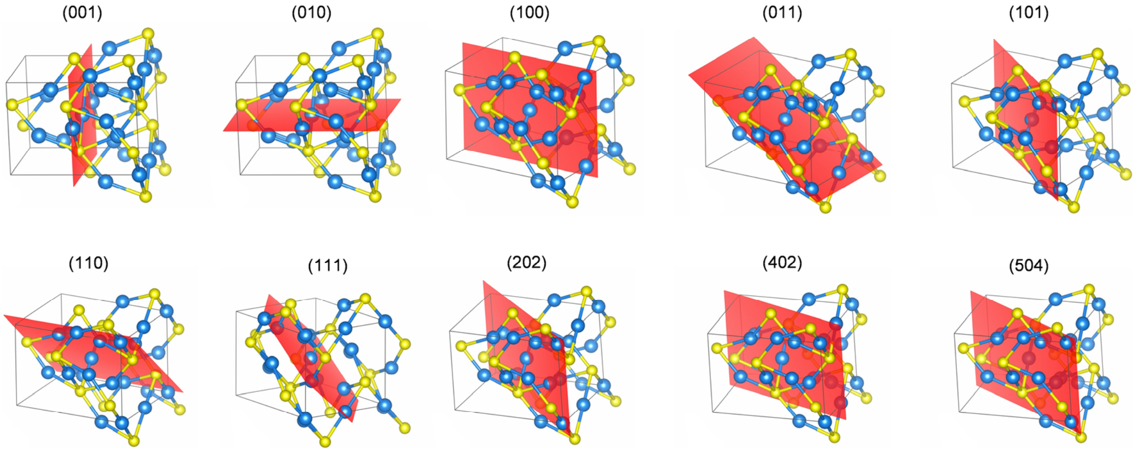

Supplementary Figure 12 Surface terminations of the Pd_3S crystal. The planes featuring different Miller indices (*hkl*) considered for the γ_s calculations in **Supplementary Table 4** are highlighted in red. Color code as in **Supplementary Figure 11**.

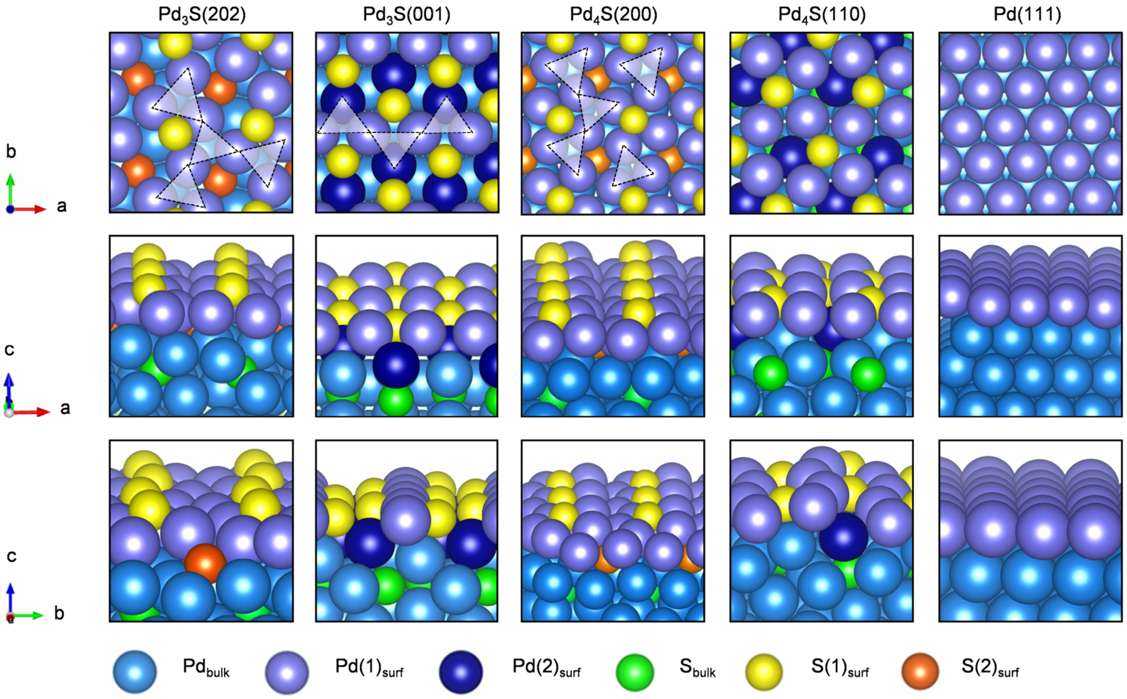

Supplementary Figure 13 Top and side views of the surface reconstructions for selected surfaces where the Pd atoms belonging to the topmost layer ($Pd(1)_{surf}$ and $Pd(2)_{surf}$) are arranged in triangular ensembles on Pd_3S(001), Pd_3S(202), and Pd_4S(200) surfaces. Note that the ensembles in Pd_3S(202) and Pd_4S(200) are composed of symmetry equivalent Pd atoms, while in the Pd_3S(001) and Pd_4S(110) surfaces two inequivalent sites can be distinguished (see **Supplementary Figure 14**).

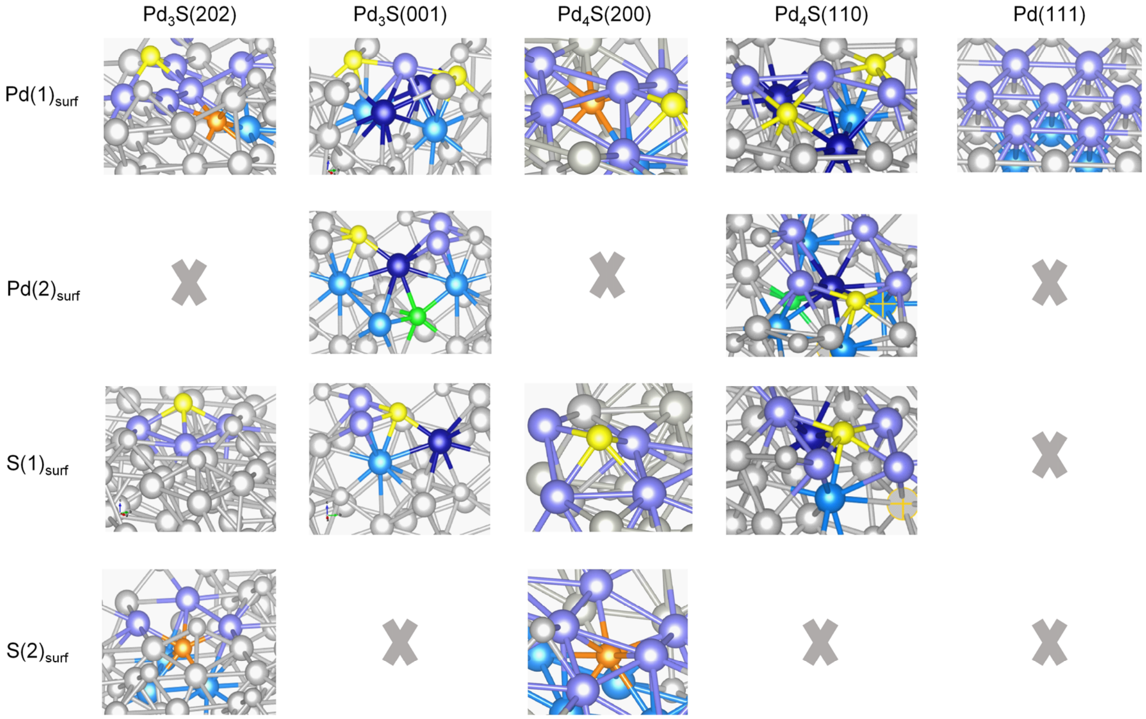

Supplementary Figure 14 Symmetry equivalent sites in the Pd_xS and Pd surfaces. 2 distinct Pd, and S sites can be distinguished. A grey 'X' denotes the lack of an equivalent site on the respective surface. On the $Pd_3S(202)$ surface, each $Pd(1)_{surf}$ is surrounded by four $Pd(1)_{surf}$, one Pd_{bulk}, one $S(1)_{surf}$, and one $S(2)_{surf}$. The $Pd_3S(001)$ surface displays two inequivalent Pd sites: the $Pd(1)_{surf}$ atom is bound to two $S(1)_{surf,}$ two $Pd(2)_{surf}$, and two Pd_{bulk}, while the $Pd(2)_{surf}$ atom is linked to two $Pd(1)_{surf}$, three Pd_{bulk}, one $S(1)_{surf}$, and one S_{bulk} atoms. On the $Pd_4S(200)$ surface, each $Pd(1)_{surf}$ is attached to four $Pd(1)_{surf}$, one Pd_{bulk}, one $S(1)_{surf}$, and one $S(2)_{surf}$. On the $Pd_4S(110)$ surface, each $Pd(1)_{surf}$ is linked to two $Pd(1)_{surf}$, two $Pd(2)_{surf}$, one Pd_{bulk} and two $S(1)_{surf}$, while each $Pd(2)_{surf}$ atom is surrounded by four $Pd(1)_{surf}$, four $Pd_{bulk,}$ one $S(1)_{surf}$ and one S_{bulk} atoms. On the Pd(111) surface, each Pd atom is attached to six $Pd(1)_{surf}$ and three Pd_{bulk} atoms. Color code as in **Supplementary Figure 13**, to highlight the closest neighbors to the considered site the remaining atoms are grayed out.

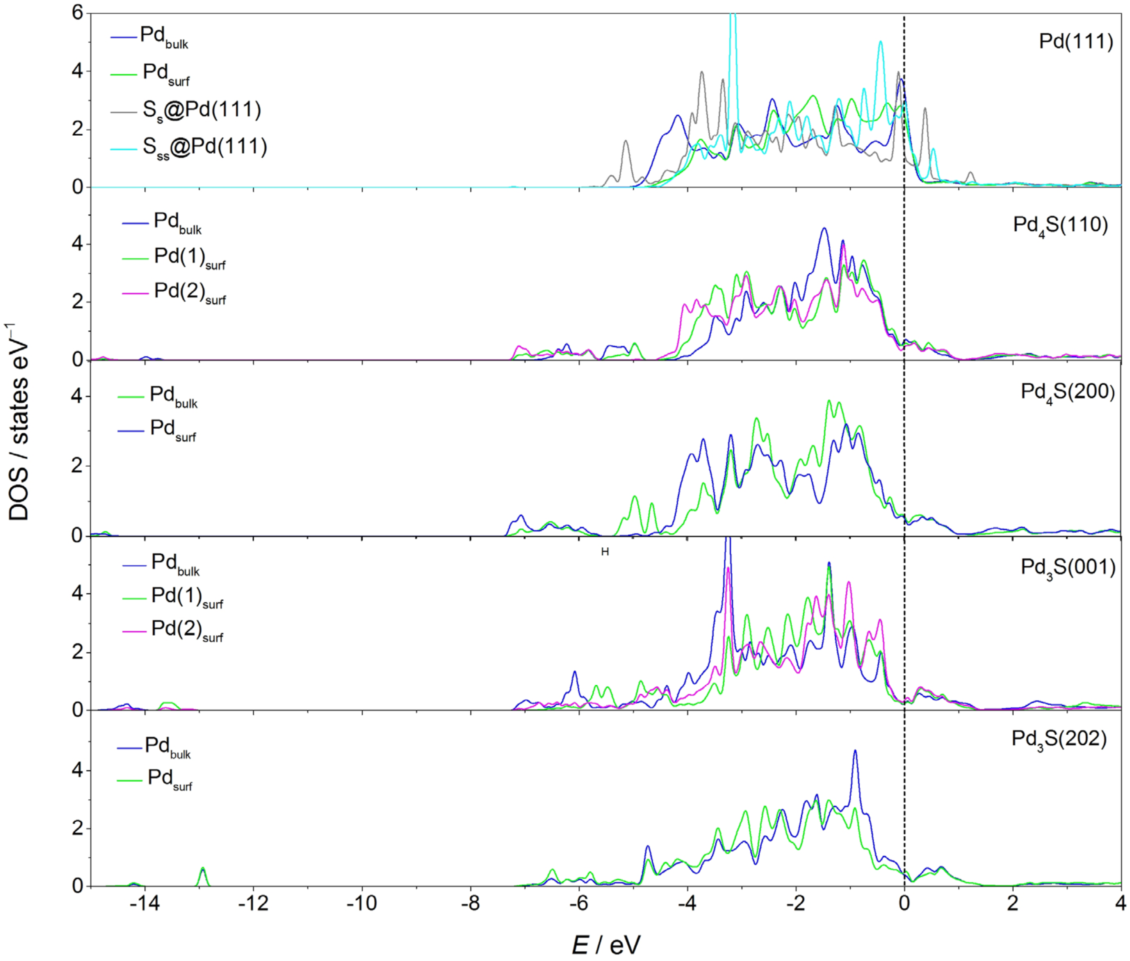

Supplementary Figure 15 Projected palladium *d*-band densities of states (DOS). The surfaces considered are the following: the bare Pd(111) and after adsorption of atomic sulfur (S_s@Pd(111)) or sulfur inclusion into subsurface site (S_{ss}@Pd(111)), Pd_4S(110), Pd_4S(200), Pd_3S(001), and Pd_3S(202). The *d*-band center (ε_d) is at –1.39, –1.85, –1.65, –1.70, –1.78, –1.91, –1.90, –1.93, and –1.80 eV on the topmost layer of Pd(111), S_s@Pd(111), S_{ss}@Pd(111), $Pd(1)_{surf}$ and $Pd(2)_{surf}$ of Pd_4S(110), $Pd(1)_{surf}$ of Pd_4S(200), $Pd(1)_{surf}$ and $Pd(2)_{surf}$ of Pd_3S(001), and $Pd(1)_{surf}$ of Pd_3S(202) surfaces, respectively. Pd atoms in the Pd_3S surfaces are attached to two S atoms, but have more Pd neighbors. The degree of sulfur poisoning is lower than the Pd_3S(001) surface, where the down-shift in ε_d is more pronounced. In fact, ε_d is located at –1.90, and –1.93 eV for $Pd(1)_{surf}$ and $Pd(2)_{surf}$ in the Pd_3S(001) surface, while it is at –1.80 eV for $Pd(1)_{surf}$ in the Pd_3S(202) surface. Interestingly, ε_d for S_s@Pd(111) system is placed at –1.85 eV, while it is located at –1.65 eV for S_{ss}@Pd(111) system, confirming the S poisoning impacts more surface than subsurface sites.

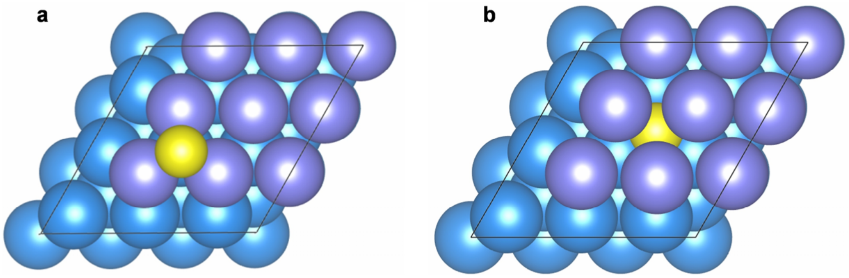

Supplementary Figure 16 Top view of **a** the adsorption of atomic sulfur (S_s@Pd(111)) and **b** sulfur inclusion into subsurface site (S_{ss}@Pd(111)) on the Pd(111) surface. Color code as in **Supplementary Figure 14**.

Supplementary Note 4 Reaction mechanism. Comprises **Supplementary Tables 5-8**, **Supplementary Figures 17-26**, and related discussion.

To obtain molecular insights on the reaction mechanism over sulfided palladium surfaces, DFT calculations for acetylene hydrogenation were conducted over the Pd(111), Pd_4S(200), Pd_4S(110), Pd_3S(001), and Pd_3S(202) surfaces. The complete reaction pathways with all respective reaction energies, activation barriers, and the adsorption energies for all intermediate states with and without zero point vibrational corrections are reported in **Supplementary Tables 5-7**, and illustrated in **Fig. 5** and **Supplementary Figures 17-20**.

Similarly from H_2 adsorption on the Pd(111) surface (E_{ads} = –0.15 eV) where the H_2 molecule lies flat on top of a Pd atom, on the Pd_3S(001), Pd_4S(200) and Pd_4S(110), H_2 sits flat on the $Pd(1)_{surf}$ since the energy difference between horizontal and vertical configurations is very small. Contrarily, on the Pd_3S(202) surface, due to thermodynamic reasons the adsorption occurs vertically on top of a $S(1)_{surf}$ atom and displays moderate endothermicity (E_{ads} = 0.01 eV). On the Pd(111) surface the reaction network starts with the exothermic H_2 dissociation (ΔE = –0.72 eV), which agrees well with the previous work[17]. The transition state is reached when the distance between the H atoms is 1.35 Å, which leads to a stable state of two H atoms coadsorbed at the fcc sites. On the other hand, molecular hydrogen dissociation takes place on adjacent Pd sites featuring endothermicity (0.18 eV) on the Pd_3S(001) surface with a barrier of 0.80 eV, exothermicity (– 0.50, and –0.02 eV) with a barrier of 0.45 and 0.65 eV on Pd_4S(110) and Pd_4S(200), respectively. The H fragments on both Pd_3S(001) and Pd_4S(110)

surfaces continue to come apart after the transition state, eventually being adsorbed on two $Pd(2)_{surf}$ atoms; on the contrary, two H atoms lie at bridge positions between two Pd atoms on the $Pd_4S(200)$ surface. Since the H_2 molecule is located on top of the $S(1)_{surf}$ atom of $Pd_3S(202)$, then its dissociation takes place on adjacent Pd and S sites with similar activation to that observed on both $CeO_2(111)$ and $In_2O_3(111)$[18,19]. This reaction is slightly exothermic (ΔE = –0.08 eV) featuring a barrier of 0.77 eV. A distance between the H atoms of 2.1 Å is required to reach the transition state, ending up to a stable state of H atoms co-adsorbed on two $S(1)_{surf}$ atoms forming two thiol groups. All hydrogen dissociation steps for all surfaces are displayed in **Supplementary Figure 18**. The reaction network is followed by the adsorption of the alkyne on the surface, which is exothermic on Pd(111), $Pd_4S(200)$, $Pd_4S(110)$, and $Pd_3S(202)$ by –2.51, –0.24, –0.28, and –0.48 eV, respectively (with respect to gas-phase acetylene and H_2), and endothermic on $Pd_3S(001)$ by 0.05 eV. Afterwards, the sequential addition of H atom follows a Horiuti–Polanyi mechanism[20]. The first hydrogen addition to the adsorbed acetylene moiety is exothermic and leads to a vinyl group ($HCCH_2$). The reaction energies for this step are –0.17, –1.04, –0.65, –0.91, and –1.21 eV and activation barriers are 0.67, 0.92, 0.66, 0.90, and 1.00 eV on Pd(111), $Pd_4S(200)$, $Pd_4S(110)$, $Pd_3S(001)$, and $Pd_3S(202)$, respectively. The second hydrogen addition can result in two competitive products, ethene (H_2CCH_2) or ethylidene ($HCCH_3$). The formation of the former features activation energies of 0.66, 0.80, 0.80, 0.30, and 1.14 eV with ΔE of –0.31, –1.08, – 1.70, – 1.35, and –0.60 eV on Pd(111), $Pd_4S(200)$, $Pd_4S(110)$, $Pd_3S(001)$, and $Pd_3S(202)$, respectively. Due to the higher activation energies of ethylidene formation, ethene formation is preferred on all surfaces. Interestingly, the comparative barrier between H_2CCH_2 and $HCCH_3$ formation on both Pd_3S surfaces is greater than those on Pd(111) and $Pd_4S(110)$ (**Fig. 5e**).

At this stage, the desired semi-hydrogenation product H_2CCH_2 could eventually undergo over-hydrogenation to ethyl (H_2CCH_3) and finally form ethane. Ethene desorption on Pd_3S surface, ΔE = 0.08 and E_a = 0.14 eV, is energetically advantageous over its further hydrogenation (0.79 and 1.06 eV), which also indicates that ethylene adsorbs on the surface *via* van der Waals attractions. Notwithstanding the H_2CCH_2 desorption on Pd_4S surface requires greater energy (0.47 and 0.22 eV), it is still favored than over-hydrogenation to ethyl which features a barrier of 0.64 and 0.55 eV. In contrast, the desorption of H_2CCH_2 is impeded on the Pd(111) surface since the energy (0.85 eV) is higher than

the third hydrogenation barrier (0.45 eV), resulting in a relatively low selectivity towards ethene. The fourth hydrogenation to ethane is exothermic on all surfaces. The reaction energies (activation barriers) for this step are –0.67 (0.44) eV, –1.00 (0.90) eV, –0.90 (1.20) eV, –1.25 (1.13) eV, and –1.20 (1.00) eV on Pd(111), Pd_4S(200), Pd_4S(110), Pd_3S(001), and Pd_3S(202), respectively. The last step is ethane desorption which occurs readily all surfaces. To probe the effects of solvation on the adsorption energies of intermediates on the supported palladium sulfide surfaces, we applied the recently developed continuum solvation model into the VASP Multigrid Continuum Model (VASP-MGCM). **Supplementary Table 8** summarizes the energies for the solvation of benzene, H_2, C_2H_2, and C_2H_4 in benzene. The solvation energies are very small, therefore similar binding energies to those reported for these molecules in **Supplementary Table 5** are expected in solution.

The second potential side reaction occurring on Pd-based catalysts, oligomer formation (*i.e.*, ethyne-ethyne coupling), along with the initial, transition, and final states, and the energy profiles on all surfaces are shown in **Supplementary Figures 19c** and **21**, and illustrated in **Fig. 5c** and **Supplementary Figure 20**. On the Pd(111) surface, the barrier for oligomerization is 1.34 eV[21]. However, the coupling barrier is higher on the Pd_xS surfaces, 1.52, 1.47, 1.75, and 1.64 eV for Pd_4S(200), Pd_4S(110), Pd_3S(001), and Pd_3S(202), respectively. This indicates how the surface anisotropy is negative to the diffusion and points to some degree of site isolation, explaining why no oligomer formation is observed during reaction over the sulfided catalysts. **Supplementary Table 5** and **6** display that the adsorption energies for all different intermediates are reduced on Pd_xS surfaces in comparison with Pd(111). These results were also extrapolated for 2-methyl-3-butyn-2-ol (C_5H_8O), and 2-methyl-3-buten-2-ol ($C_5H_{10}O$) on all Pd_xS surfaces. The optimized structures of these molecules bound to all surfaces are shown in **Supplementary Figure 22**. The adsorption energy of C_5H_8O molecule (with respect to gas-phase molecule) is –1.92, –0.16, –0.66, –0.01, and –0.10 eV on Pd(111), Pd_4S(200), Pd_4S(110), Pd_3S(001), and Pd_3S(202) surfaces, respectively, while the values for the $C_5H_{10}O$ molecule are –0.73, –0.04, –0.22, 0.10, and 0.05 eV, respectively. This behavior has been rationalized through analysis of the *d*-band center[22,23], which evidenced a shifted away from the Fermi level moving from Pd to Pd_4S and Pd_3S (**Supplementary Figures 15** and **26**). Ultimately, the introduction of sulfur decreased the adsorption energies of all intermediates.

Supplementary Table 5 Reaction energy and corresponding activation barrier for the elementary steps in acetylene hydrogenation on Pd_3S(202), Pd_3S(001), Pd_4S(200), Pd_4S(110), and Pd(111) surfaces. The steps are color coded black - desired; red - ethylidene ($HCCH_3$) formation; blue - ethyl (H_2CCH_3) or ethane (H_3CCH_3) formation; green - ethylene (H_2CCH_2) or ethane (H_3CCH_3) desorption; brown - oligomerization (C_4H_4). Adsorbed species are denoted with asterisks. The oligomerization energies are calculated with respect to two acetylene and two H_2 molecules in the gas phase.

No.	Elementary step	Pd_3S(202)		Pd_3S(001)		Pd_4S(200)		Pd_4S(110)		Pd(111)	
		ΔE / eV	E_a / eV	ΔE / eV	E_a / eV	ΔE / eV	E_a / eV	ΔE / eV	E_a / eV	ΔE / eV	E_a / eV
R1	H_2* → 2H*	–0.08	0.77	0.18	0.80	–0.02	0.65	–0.50	0.45	–0.72	-
R2	HCCH* + 2H* → $HCCH_2$* +H*	–1.21	1.00	–0.91	0.90	–1.04	0.92	–0.65	0.66	–0.17	0.67
R3	$HCCH_2$* + H* → H_2CCH_2*	–0.60	1.14	–1.35	0.30	–1.08	0.80	–1.70	0.80	–0.31	0.66
R4	$HCCH_2$ + H* → $HCCH_3$*	–0.33	1.30	–0.18	0.45	–0.12	0.93	–0.55	0.85	0.05	0.72
R5	H_2CCH_2* → H_2CCH_2	0.08	-	0.14	-	0.22	-	0.47	-	0.85	-
R6	H_2CCH_2* + H* → H_2CCH_3*	–0.32	0.79	–0.30	1.06	–0.41	0.55	–0.30	0.64	–0.20	0.45
R7	H_2CCH_3* + H* → H_3CCH_3*	–1.20	1.00	–1.25	1.13	–1.00	0.90	–0.90	1.20	–0.67	0.44
R8	H_3CCH_3* → H_3CCH_3	0.00	-	0.00	-	0.02	-	0.04	-	0.05	-
R9	HCCH* + HCCH* → C_4H_4*	0.03	1.64	–0.05	1.75	–0.07	1.52	–0.60	1.47	0.09	1.34

Supplementary Table 6 Zero point vibrational corrected reaction energy and corresponding activation barrier for the elementary steps in acetylene hydrogenation on $Pd_3S(202)$, $Pd_3S(001)$, $Pd_4S(200)$, $Pd_4S(110)$, and Pd(111) surfaces. The steps are color coded black - desired; red - ethylidene ($HCCH_3$) formation; blue - ethyl (H_2CCH_3) or ethane (H_3CCH_3) formation; green - ethylene (H_2CCH_2) or ethane (H_3CCH_3) desorption; brown - oligomerization (C_4H_4). Adsorbed species are denoted with asterisks. The oligomerization energies are calculated with respect to two acetylene and two H_2 molecules in the gas phase.

No.	Elementary step	$Pd_3S(202)$		$Pd_3S(001)$		$Pd_4S(200)$		$Pd_4S(110)$		Pd(111)	
		ΔE / eV	E_a / eV	ΔE / eV	E_a / eV	ΔE / eV	E_a / eV	ΔE / eV	E_a/ eV	ΔE / eV	E_a / eV
R1	$H_2^* \rightarrow 2H^*$	–0.10	0.50	0.03	0.53	–0.17	0.38	–0.60	0.17	–0.85	-
R2	$HCCH^* + 2H^* \rightarrow HCCH_2^* + H^*$	–1.11	0.91	–0.48	1.20	–0.87	0.68	–0.15	0.93	0.27	0.94
R3	$HCCH_2^* + H^* \rightarrow H_2CCH_2^*$	–0.45	1.09	–1.17	0.36	–0.95	0.82	–1.56	0.77	–0.11	0.72
R4	$HCCH_2 + H^* \rightarrow HCCH_3^*$	–0.26	1.23	–0.09	0.41	–0.08	0.91	–0.25	0.84	0.24	0.70
R5	$H_2CCH_2^* \rightarrow H_2CCH_2$	–0.25	-	–0.13	-	–0.06	-	0.21	-	0.58	-
R6	$H_2CCH_2^* + H^* \rightarrow H_2CCH_3^*$	–0.26	0.92	–0.11	1.20	–0.26	0.74	0.07	0.79	–0.11	0.25
R7	$H_2CCH_3^* + H^* \rightarrow H_3CCH_3^*$	–1.09	1.13	–1.27	1.19	–0.85	1.02	–0.73	1.29	–0.52	0.25
R8	$H_3CCH_3^* \rightarrow H_3CCH_3$	–0.03	-	0.14	-	–0.01	-	0.06	-	0.15	-
R9	$HCCH^* + HCCH^* \rightarrow C_4H_4^*$	0.13	1.61	0.12	1.70	–0.07	1.39	–0.60	1.30	0.12	1.29

Supplementary Table 7 Comparative adsorption energies of the alkyne hydrogenation intermediates on the Pd_3S(202), Pd_3S(001), Pd_4S(200), Pd_4S(110), and Pd(111) surfaces with respect to the free alkyne and H_2 molecules and a reference surface.

Intermediate	E_{ads} / eV Pd_3S(202)	E_{ads} / eV Pd_3S(001)	E_{ads} / eV Pd_4S(200)	E_{ads} / eV Pd_4S(110)	E_{ads} / eV Pd(111)
H_2*	0.01	0.01	0.02	–0.05	–0.15
2H*	–0.07	0.19	0.00	–0.10	–0.87
HCCH*	–0.48	0.05	–0.24	–0.28	–2.51
$HCCH_2$*	–1.69	–0.86	–1.28	–0.93	–2.68
$HCCH_3$*	–2.02	–1.05	–1.41	–1.48	–2.64
H_2CCH_2*	–2.29	–2.20	–2.36	–2.64	–2.99
H_2CCH_3*	–2.60	–2.49	–2.77	–2.94	–3.18
H_3CCH_3*	–3.79	–3.74	–3.78	–3.83	–3.85
C_4H_4*	–0.98	0.05	–0.07	–2.20	–4.94
C_5H_8O*	–0.10	–0.01	–0.16	–0.66	–1.92
$C_5H_{10}O$*	0.05	0.10	–0.04	–0.22	–0.73

Supplementary Table 8 The energies for the solvation of benzene (C_6H_6), H_2, C_2H_2, and C_2H_4 molecules in benzene.

	C_6H_6	HCCH	H_2CCH_2	H_2
E_{sol} / eV	0.02	–0.03	0.01	0.01

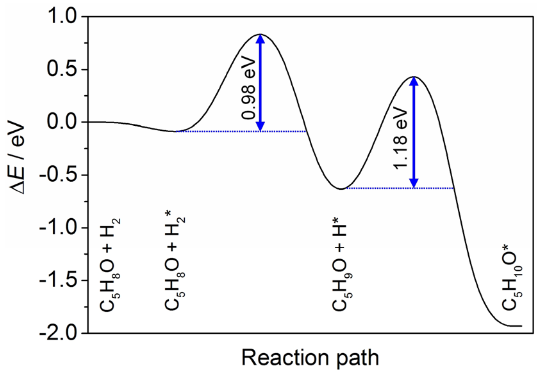

Supplementary Figures 17 Energy profile of the semi-hydrogenation of 2-methyl-3-butyn-2-ol on the Pd_3S(202) surface. Adsorbed species are denoted by asterisks. The activation energy for the first hydrogen addition (0.98 eV) is comparable with that computed for acetylene (1.00 eV), while E_a for the second hydrogenation is 1.18 eV compared to 1.14 eV for acetylene. These results further validate the use of acetylene as a surrogate for unravelling performance differences over the catalysts studied.

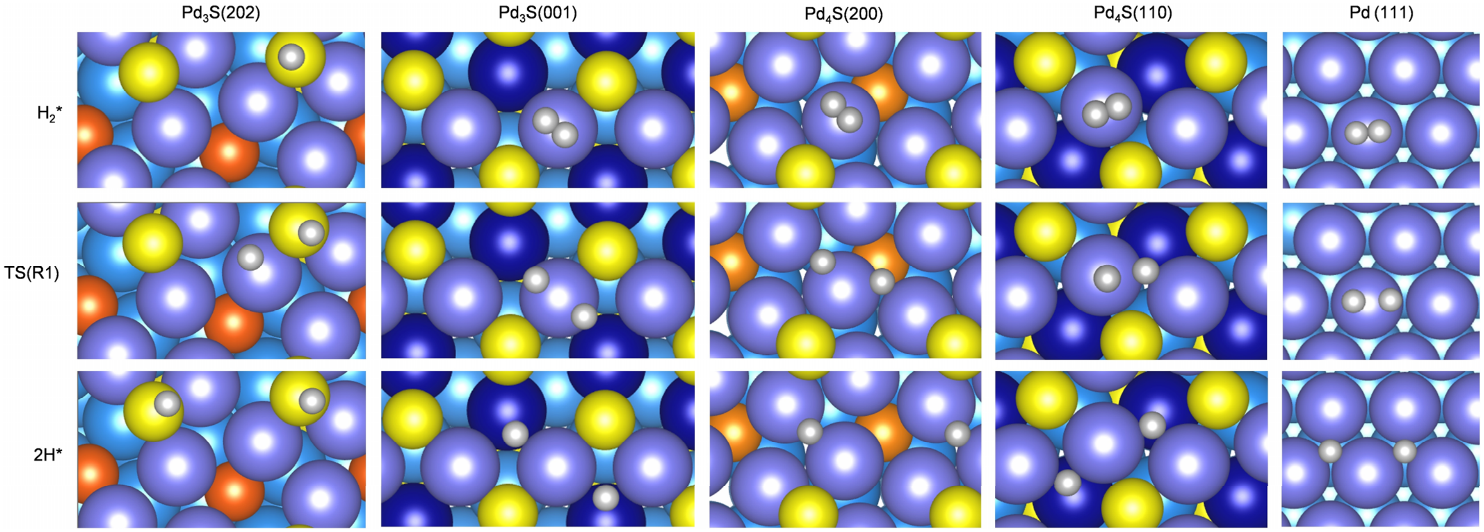

Supplementary Figure 18 Top view of the initial (H_2^*), transition (TS(R1)), and final ($2H^*$) states for the dissociation of hydrogen on the $Pd_3S(202)$, $Pd_3S(001)$, $Pd_4S(200)$, $Pd_4S(110)$, and Pd(111) surfaces. Color code as in **Supplementary Figure 13**, hydrogen (white).

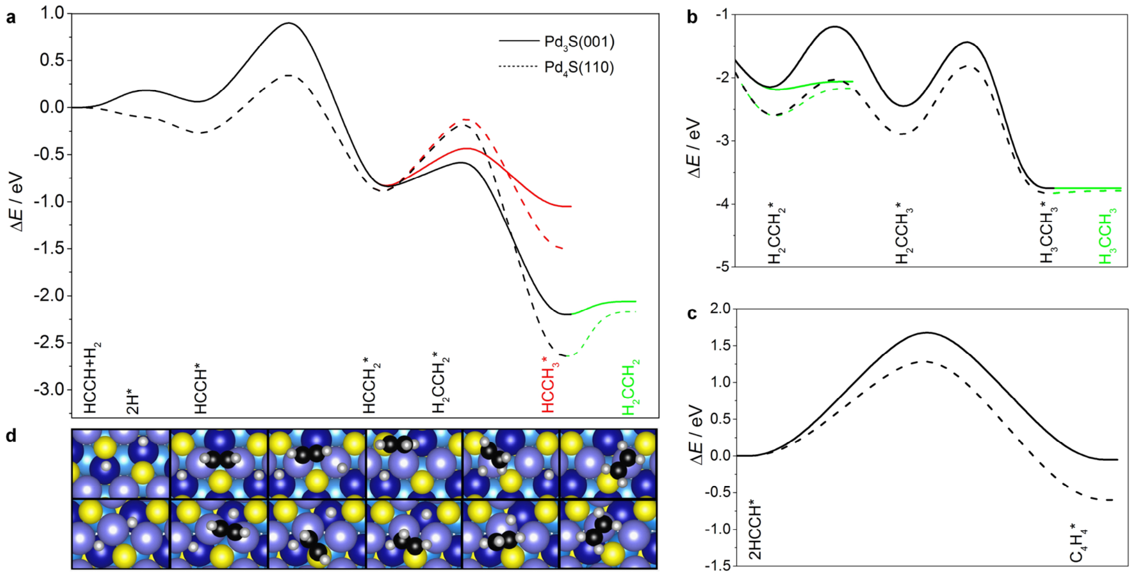

Supplementary Figure 19 Energy profiles of the **a** semi-hydrogenation, **b** over-hydrogenation, and **c** oligomerization of acetylene on the $Pd_4S(001)$ and $Pd_3S(001)$ surfaces. Adsorbed species are denoted by asterisks. The competitive formation of ethylidene is shown in red, and ethene and ethane desorption in green. **d** Top view of the DFT-optimized adsorption configuration of the reaction intermediates and transition states (TS) with respect to **Supplementary Table 5** on $Pd_3S(001)$ (top) and $Pd_4S(110)$ (bottom) surfaces. Color code as in **Supplementary Figure 18**, carbon (black).

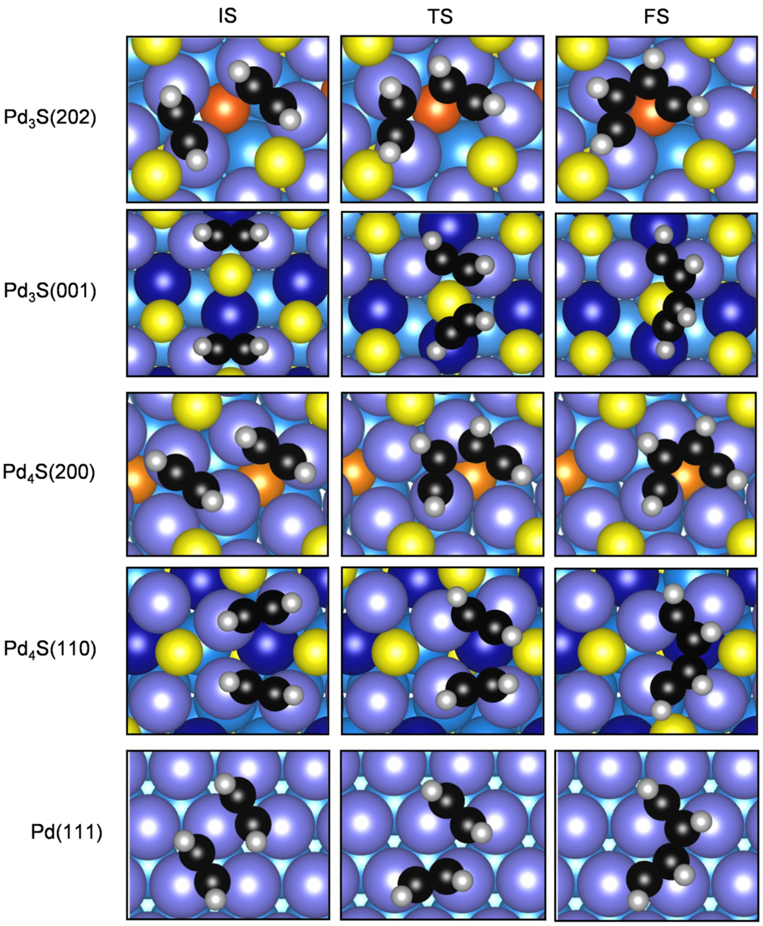

Supplementary Figure 20 Configuration of the initial ($2C_2H_2^*$), transition (TS(R8)), and final ($C_4H_4^*$) states of the oligomerization (*i.e.*, ethyne-ethyne coupling) side-path over the surfaces. Color code as in **Supplementary Figure 18**, carbon (black).

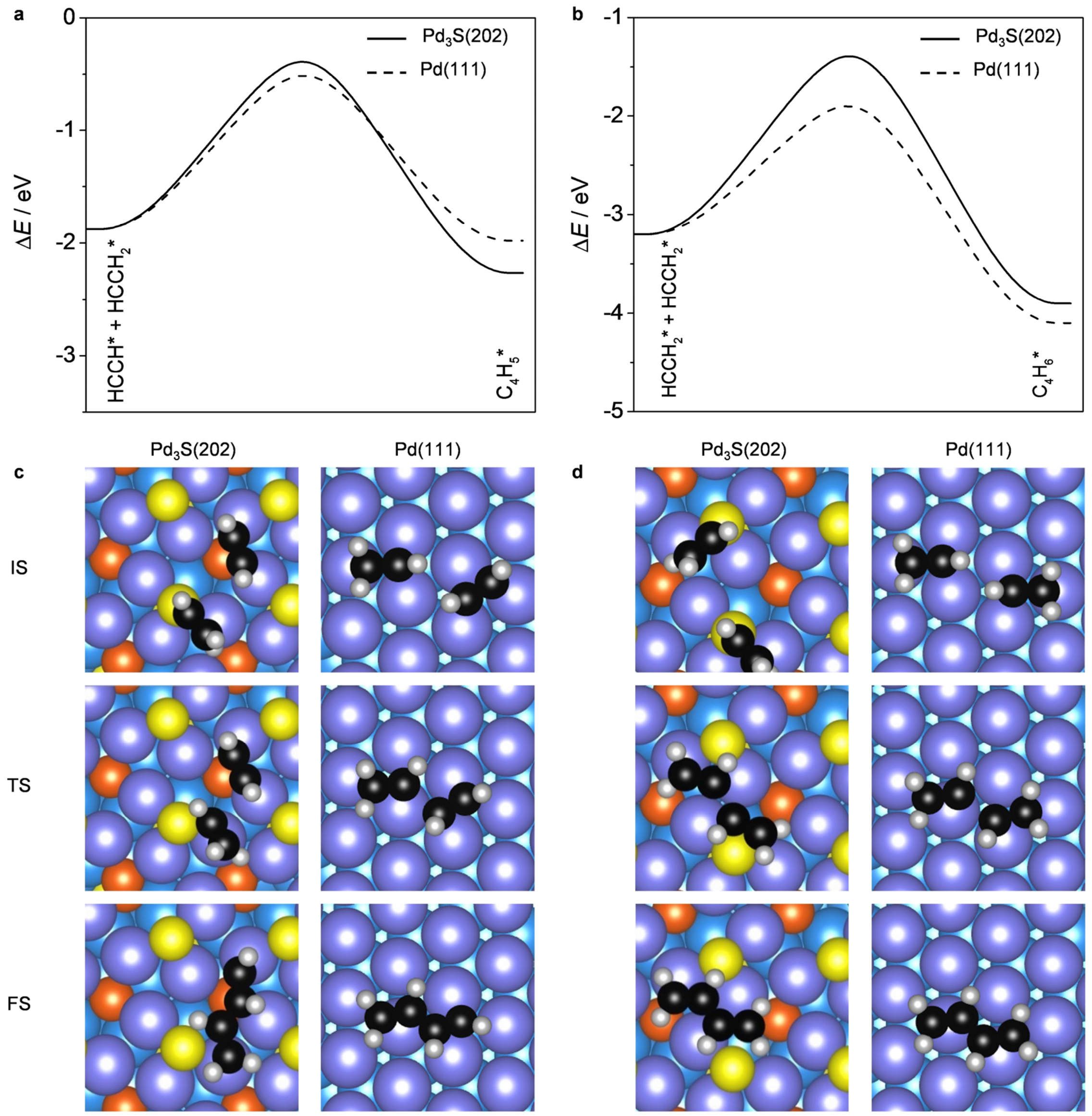

Supplementary Figure 21 a,**b** Energy profiles for alternative oligomerization pathways and **c**,**d** top view of the DFT-optimized adsorption configurations of the initial (IS), transition (TS), and final (FS) states on the $Pd_3S(202)$ and Pd(111) surfaces considering the following reactions: HCCH* + $HCCH_2$* → C_4H_5* (**a**,**c**), and $HCCH_2$* + $HCCH_2$* → C_4H_6* (**b**,**d**). Color code as in **Supplementary Figure 18**, carbon (black).

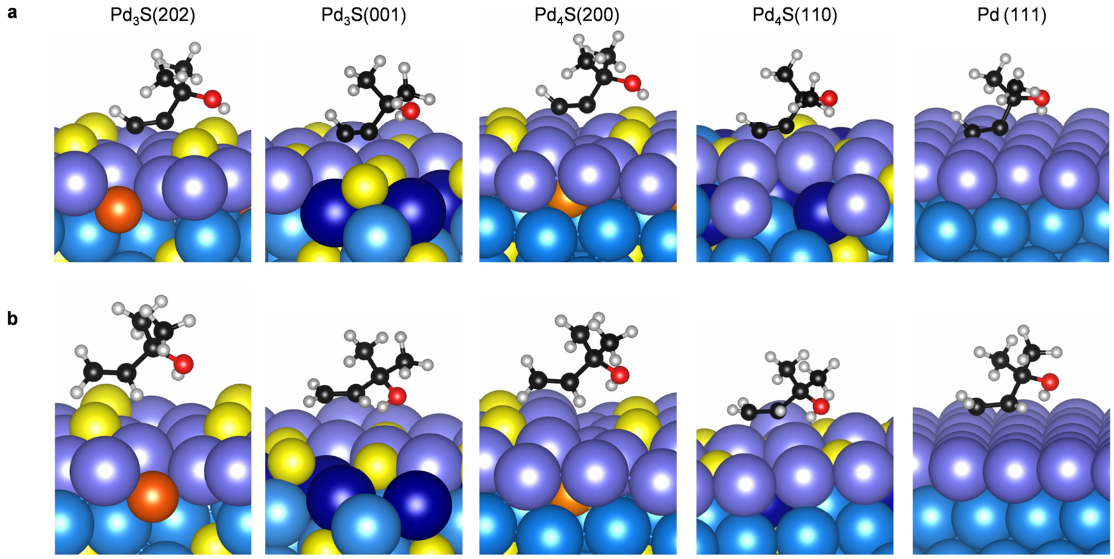

Supplementary Figure 22 Side views of the adsorption of the 2-methyl-3-butyn-2-ol and 2-methyl-3-buten-2-ol molecules on the palladium surfaces. Color code as in **Supplementary Figure 20**, oxygen (red).

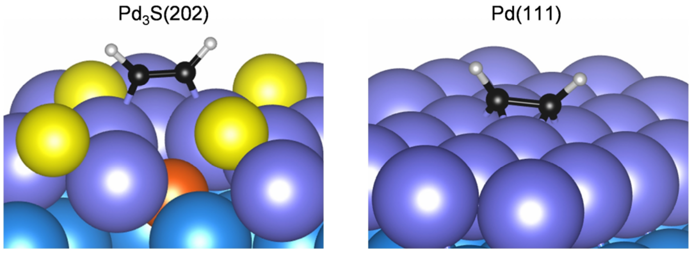

Supplementary Figure 23 Side views of the adsorption of an acetylene (C_2H_2) molecule on the palladium sulfide and palladium surfaces. Color code as in **Supplementary Figure 20**.

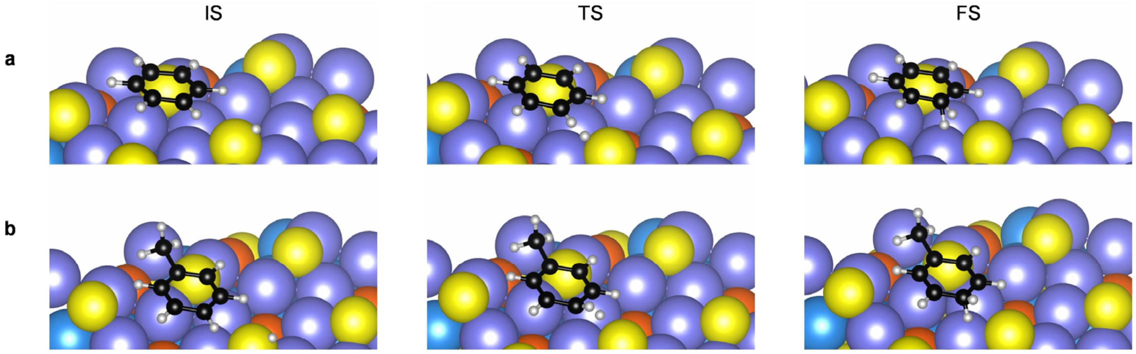

Supplementary Figure 24 Side views of the DFT-optimized adsorption configuration of the initial, transition, and final states for the first hydrogenation of benzene and toluene on the $Pd_3S(202)$ surface. Color code as in **Supplementary Figure 20**.

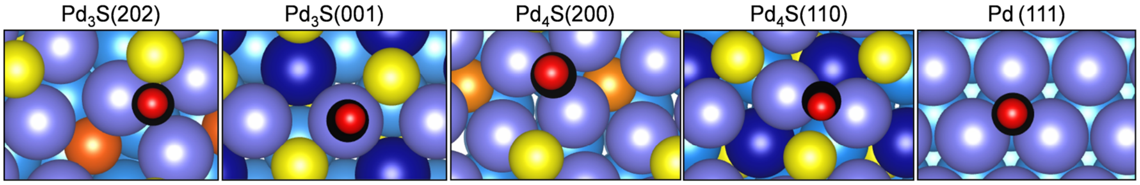

Supplementary Figure 25 Top views of the adsorption of a CO molecule on the palladium sulfide and palladium surfaces. Color code as in **Supplementary Figure 20**. The calculated adsorption energies, ΔE, indicate that CO should adsorb on all surfaces with the following values: Pd(111), –1.85 eV; $Pd_4S(200)$, –0.57 eV; $Pd_3S(202)$, –0.65 eV; and $Pd_3S(001)$, –0.52 eV.

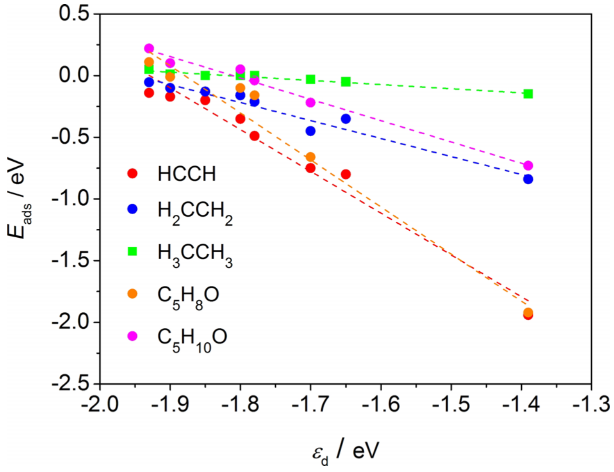

Supplementary Figure 26 Adsorption energies of C_2H_2, C_2H_4, C_2H_6, C_5H_8O, and $C_5H_{10}O$ species as a function of the *d*-band center (ε_d) of Pd atoms on the topmost surface layer (**Supplementary Figure 15**). These results highlight how the shift of the *d*-band center upon sulfur incorporation or doping leads to a decrease of the adsorption energies of all reaction intermediates on Pd_xS.

Supplementary References

1. Makov, G. & Payne, M. C. Periodic boundary conditions in ab initio calculations. *Phys. Rev. B* **51**, 4014–4022 (1995).
2. Kresse, G. & Furthmüller, J. Efficient iterative schemes for ab initio total-energy calculations using a plane-wave basis set. *Phys. Rev. B* **54**, 11169–11186 (1996).
3. Kresse, G. & Furthmüller, J. Efficiency of ab-initio total energy calculations for metals and semiconductors using a plane-wave basis set. *Comput. Mater. Sci.* **6**, 15–50 (1996).
4. Hammer, B., Hansen, L. B. & Nørskov, J. K. Improved adsorption energetics within density-functional theory using revised Perdew-Burke-Ernzerhof functionals. *Phys. Rev. B* **59**, 7413–7421 (1999).
5. Blöchl, P. E. Projector augmented-wave method. *Phys. Rev. B* **50**, 17953–17979 (1994).
6. Grimme, S. Semiempirical GGA-type density functional constructed with a long-range dispersion correction. *J. Comput. Chem.* **27**, 1787–1799 (2006).
7. Bučko, T., Hafner, J., Lebègue, S. & Ángyán, J. G. Improved dscription of the structure of molecular and layered crystals: ab initio DFT calculations with van der Waals corrections. *J. Phys. Chem. A* **114**, 11814–11824 (2010).
8. Almora-Barrios, N., Carchini, G., Błoński, P. & López, N. Costless derivation of dispersion coefficients for metal surfaces. *J. Chem. Theory Comput.* **10**, 5002–5009 (2014).

9. Røst Erling, V. E. The crystal structure of the high temperature phase Pd_3S. *Acta Chem. Scand.* **22**, 819–826 (1968).
10. Røst, F. G. a. E. The crystal structures of Pd_4Se and Pd_4S. *Acta. Crsytallogr* **15**, 3 (1962).
11. Miller, J. B., Alfonso, D. R., Howard, B. H., O'Brien, C. P. & Morreale, B. D. Hydrogen dissociation on Pd_4S surfaces. *J. Phys. Chem. C* **113**, 18800–18806 (2009).
12. Henkelman, G. & Jónsson, H. Improved tangent estimate in the nudged elastic band method for finding minimum energy paths and saddle points. *J. Chem. Phys.* **113**, 9978–9985 (2000).
13. Henkelman, G., Uberuaga, B. P. & Jónsson, H. A climbing image nudged elastic band method for finding saddle points and minimum energy paths. *J. Chem. Phys.* **113**, 9901–9904 (2000).
14. Hu, R., Gao, M. C., Doğan, Ö. N., King, P. & Widom, M. Thermodynamic modeling of the Pd–S system supported by first-principles calculations. *Calphad* **34**, 324–331 (2010).
15. Ivantchev, S., Kroumova, E., Madariaga, G., Pérez-Mato, J. M. & Aroyo, M. I. SUBGROUPGRAPH: a computer program for analysis of group-subgroup relations between space groups. *J. Appl. Crystallogr.* **33**, 1190–1191 (2000).
16. Henkelman, G., Arnaldsson, A. & Jónsson, H. A fast and robust algorithm for Bader decomposition of charge density. *Comput. Mater. Sci.* **36**, 354–360 (2006).
17. Lopez, N., Łodziana, Z., Illas, F. & Salmeron, M. When Langmuir is too simple: H_2 dissociation on Pd(111) at high coverage. *Phys. Rev. Lett.* **93**, 146103 (2004).
18. Albani, D. *et al.* Semihydrogenation of acetylene on indium oxide: proposed single-ensemble catalysis. *Angew. Chem. Int. Ed.* **56**, 10755–10760 (2017).
19. García-Melchor, M. & López, N. Homolytic products from heterolytic paths in H_2 dissociation on metal oxides: the example of CeO_2. *J. Phys. Chem. C* **118**, 10921–10926 (2014).
20 I. Horiuti, Polanyi, M. Exchange reactions of hydrogen on metallic catalysts. *Trans. Faraday Soc.* **30**, 1164–1172 (1934).
21. García-Mota, M., Bridier, B., Pérez-Ramírez, J. & López, N. Interplay between carbon monoxide, hydrides, and carbides in selective alkyne hydrogenation on palladium. *J. Catal.* **273**, 92–102 (2010).
22. Hammer, B. & Norskov, J. K. Why gold is the noblest of all the metals. *Nature* **376**, 238–240 (1995).
23. Hammer, B. & Nørskov, J. K. Electronic factors determining the reactivity of metal surfaces. *Surf. Sci.* **343**, 211–220 (1995).
